# In vitro assessment of metabolic profile of *Enterococcus* strains of human origin

**DOI:** 10.1186/s43141-019-0009-0

**Published:** 2019-11-25

**Authors:** Ashlesha Bhagwat, Uday S. Annapure

**Affiliations:** Department of Food Engineering and Technology, Institute of Chemical Technology, Mumbai, 400019 India

**Keywords:** *Enterococcus*, Enzymes, BA, BSH, CLA, Antioxidant

## Abstract

**Background:**

In the present study, previously isolated, safe, and avirulent enterococci strains were exploited for their metabolic profile (Bhagwat et al., Asian J Pharm Clin Res 12: 2019).

**Results:**

Thirteen enterococci strains of human origin produced important enzymes like amylase (0.5–0.7 mg ml^−1^), protease (192–264 mg ml^−1^), lipase (8–10 mg ml^−1^), bile salt hydrolase, conjugated linoleic acid (CLA), and lactic acid (highest 12 mg ml^−1^), thus implicating potential attributes of starter cultures in food and dairy industry. Biogenic amines like arginine and tryptamine were produced after 4 days above 25 °C. Castor oil (highest yield 60 μg ml^−1^) and sunflower oil (highest yield 48 μg ml^−1^) both proved to be excellent sources of CLA production. Reduction assays using FRAP, ABTS (above 83%), and DPPH (30–50%) revealed excellent radical scavenging properties of cell-free supernatants of *Enterococcus* strains.

**Conclusion:**

The results implicate the future potential of application enterococci for therapeutic purpose as well as the food industry.

## Background

The gut microbiota is very diverse and has varied metabolic activities. These activities are useful as well as harmful to the host. The potential of the probiotic organism depends on the beneficial secondary metabolites produced by them. They are known to produce various enzymes like β-galactosidase, proteases, lipases, and bacteriocins that possess antimicrobial activity. β-galactosidase helps in digesting the lactose which in turn helps in healing the lactose-intolerant people. Proteases and lipases help in the formation of biofilms, in turn, improving the process of pathogen exclusion. These enzymes also help in reducing hypertension [2, 3]. Hence, the screening of probiotics considers metabolite production in their selection criteria [4, 5].

Probiotic strains are also widely used as starter cultures in the food and dairy industry for, e.g., cheese ripening, wine, beer making. Several lactic acid bacteria are known to produce biogenic amines (BA). These amines are produced from the decarboxylase activity of the organism. They are organic, basic, and nitrogenous toxic substances that produce toxic amines from proteinaceous substances. Some of the important amines are putrescine, cadaverine, histamine, and agmatine and produced from ornithine, lysine, histidine, arginine, and tyrosine, respectively. The presence of BA in foods in higher concentrations indicates undesirable microbial activity in food. BA are present in the human body in small amounts and involved in biological processes like an immune response, synaptic transmission, and cell growth. But, the consumption of BA in large amounts can be harmful. Hence, it is necessary to prevent its formation in food products. The lactic acid bacteria produce BA under specific temperature and conditions; hence, a strain should only be passed if the strain does not produce the amine at the required temperature [6, 7].

Conjugated linoleic acids (cis-9, trans-11-CLA and trans-10, cis-12-CLA) are isomers naturally found in milk, tissue-containing fat. It is a biogenic isomer essentially a mixture of geometric and positional isomers of linoleic acid (C18:2). Recent studies have reported their multiple properties like anticarcinogenic, anti-obesity, and immune-modulatory activities [8–11]. Owing to their numerous health benefits, there has been an increase in research related to producing these isomers with the help of human-derived starter cultures in the food industry. Different strains of lactobacilli, bifidobacteria, and *Propionibacterium* have revealed their bioactive fatty acid-producing properties in milk or synthetic media [12].

Today, enterococci have created interest in the field of probiotics. The use of enterococci in foods is now a cause of concern due to the presence of virulent enterococci in nosocomial infections. According to FAO and WHO, the possession of virulent genes does not generalize the organism to be pathogenic. Enterococci like *E. faecium* M74 and *E. faecium* SF68 are in commercial use in animal feed and have proved their probiotic importance [13, 14]. Currently, there is a need to assess the specific bioactivities of microorganisms in intestinal microbiology. Hence, the present investigation attempted at screening of enterococci strains for their beneficial attributes. The screening will also help to detect industrially important enzymes of the food industry.

## Methods

### Bacterial strains and growth conditions

Healthy human vagina and gastrointestinal tract are important sources of gut-stabilizing beneficial bacteria. Maternal microbiota is the foundation of gut development of the neonate. Thirteen *Enterococcus* strains used in the study were isolated from the healthy human vagina and fresh meconium of the neonates and identified by 16S rRNA sequencing and submitted to NCBI GenBank under accession numbers KX830968–KX830982. The study was approved by the Ethics Committee of Lokmanya Tilak General Municipal Corporation Hospital, Mumbai, India. The non-virulent strains were previously characterized for in vitro probiotic aspects [1]. The study involved strains indigenous to human source as human origin strains are preferred for probiotic use according to the criterion laid down by Food and Drug Administration (FDA). The strains were grown in MRS broth at 37 °C aerobically for 18 h, preserved in 40% glycerol stocks at − 20 °C and sub-cultured periodically. All the strains were sub-cultured in MRS broth before use for each assay. The commercially well-known probiotic strain *Lactobacillus casei* Shirota (*L. casei* YIT 9029) was used as a reference strain for comparison for all the assays.

### Starch, Tween 80, and cellulose hydrolysis

The amylolytic and cellulolytic activity was checked by spot inoculating the strain on MRS agar supplemented with 1% starch and 1% carboxy methyl cellulose following incubation at 30 °C, for 4–5 days. Lugol’s solution was added and spread all over the plate and the halo created due hydrolysis of starch and cellulose was measured [15]. The lipolytic activity was checked by streaking culture on MRS medium supplemented with 1% Tween 80 following incubation at 30 °C for 4–5 days. Precipitation with a halo around the colonies indicated lipolytic activity [16].

### Gelatinase, urease, and oxidase activity

The culture strain was stab inoculated into gelatin medium (peptone 5 g l^−1^, beef extract 3 g l^−1^, gelatin 120 g l^−1^) and incubated at 37 °C for 5 days. The tubes were then kept at 10 °C for 15–20 min and observed for gelatin liquefaction by tilting the tube [17]. The urease activity was checked by inoculating strains in Christenson’s urea agar plates with phenol red as the indicator. A pink zone around the colony indicated urease activity [18]. For oxidase activity, a well-isolated colony of the test culture was spread on the oxidase disc. A blue color change within 15–30 s was reported as a positive reaction for the presence of oxidase enzyme [19, 20].

### Hippurate and esculin hydrolysis

A hippurate impregnated disc was added to BHI broth with the test culture and incubated at 37 °C for 24 h. Two milliliters of the supernatant obtained by centrifugation was mixed with 2 ml of ferric chloride reagent. Precipitation persisting for more or equal to 10 min indicated the presence of hippurate enzyme [21]. For esculin hydrolysis, 18-h-old test culture was swabbed and esculin discs were embedded on bile containing agar, incubated at 37 °C for 72 h. Black precipitation around the disc indicated esculinase activity [22].

### Deconjugation of bile salts

For this test, MRS agar supplemented with 0.5% taurodeoxycholic acid and 0.37% calcium chloride was poured into petri plates and placed in anaerobic jars for at least 72 h before use. Wells were cut and inoculated with 10 μl 18-h-old culture. The plates were then incubated anaerobically at 37 °C for 72 h. Highly active strains precipitated bile in 48 h. Precipitation halo around the colonies was measured for bile deconjugation activity. *Lactobacillus casei* Shirota and *E. coli* ATCC 8739 were used as positive control and negative control, respectively [23].

### Production of biogenic amines

An improved method was applied to check the decarboxylase activity of the strains. The decarboxylase activity was checked by inoculating various amino acids in the Moeller’s decarboxylase medium (Sigma Chemicals, USA). The amino acid discs of ornithine, lysine, tyrosine, proline, histidine, arginine, and serine were used for detecting biogenic amine activity. The disc was inoculated into the broth and overlaid with paraffin oil and incubated at 10 °C, 20 °C, 25 °C, and 37 °C for 10 days, respectively. The color change from yellow to purple was scored as positive for the amine production [24].

### Protease, amylase, and lipase activity

The protease activity was evaluated by using casein as the substrate as described by Nguyen et al. One unit of protease activity was determined by the amount of enzyme required to release TCA-soluble fragment giving blue color equivalent to 1 μg of tyrosine [25].

The alpha-amylase activity of enterococci was determined by DNSA (dinitro salicylic acid) method. The supernatant of cells grown in BHI medium was obtained by centrifugation (8000 rpm, 15 min, 4 °C). The crude supernatant was then used for determining the amylase activity. One unit of an enzyme is defined as the amount of enzyme that allows the hydrolysis of 10 mg of starch for 15 min under given conditions [26].

For lipase assay, test culture was grown in MRS broth supplemented with 1% tributyrin for 48 h at 37 °C. The culture was centrifuged (8000 rpm, 10 min, 4 °C), resuspended in 0.05 M of PBS (pH 6.5), and sonicated to obtain cell-free supernatant. The substrate used for analysis consisted of 5% polyvinyl alcohol mixed with 20 ml of olive oil and 80 ml of double distilled water. The mixture was stirred overnight and sterilized prior to use. Then, 2.5 ml of the substrate with 2 ml of buffer and 0.5 ml of supernatant was incubated at 37 °C for 20 min. The reaction was terminated by adding 10 ml acetone. Distilled water was used as a control in the assay. A unit of lipase enzyme can be defined as the amount of 0.05 M NaOH required to neutralize the released after hydrolysis of fatty acids per 1 ml of enzyme per minute [27].

### β-galactosidase activity

The cell pellet was obtained by centrifuging 18-h-old culture at 6000 rpm, 4 °C for 10 min. The pellet was washed twice, centrifuged again, and resuspended in PBS (0.01 M, pH 7). The cells were sonicated or homogenized using mortar and pestle at 4 °C. The resulting supernatant was then filtered to obtain a cell-free supernatant. A single ONPG disc was added to 1 ml of the supernatant and incubated for 30 min at 37 °C. The reaction was stopped by adding 2 ml Na_2_CO_3_ (0.6 M). β-galactosidase cleaves ONPG to release ortho-nitrophenol. Its content was measured by noting the absorbance at 420 nm for 0, 30, 60, and 90 min interval time [28].

### Conjugated linoleic acid production

An improved rapid method was employed for CLA producing enterococci. Briefly, test cultures were inoculated in MRS broth supplemented with free linoleic acid (0.5 mg ml^−1^) and castor oil (1%) and sunflower oil (1%) followed by incubation at 37 °C for 48 h. One percent Tween 80 was added to each tube for production of lipase and free availability of linoleic acid from the substrates. The culture broth (1 ml) was centrifuged at 20,800 g for 1 min and the pellet was discarded. The supernatant was mixed with 2 ml of isopropanol, vortexed and rested for 3 min, following acid extraction by adding 1.5 ml hexane. The CLA content was determined spectrophotometrically at 233 nm by dispensing 230 μl of fat-soluble hexane layer in microtiter plate [29].

### Lactic acid production

Briefly, 25 ml of test broth culture was titrated with 0.1 N NaOH using 1 ml phenolphthalein as the indicator until the color change to pink. The lactic acid content was calculated according to AOAC standards (1 ml NaOH = 90.08 mg of lactic acid) [15, 30].

### Determination of reduction activity by ferric reducing antioxidant power (FRAP)

The culture was centrifuged at 8000 rpm for 10 min at 4 °C. The cell-free supernatant was obtained and filtered through 0.22 μ Merck-Millipore filter and used for antioxidant assays. Briefly, 0.5 ml of cell-free supernatant was mixed with 0.5 ml of 0.2 M sodium phosphate buffer (pH 6.6) and 0.5 ml of 1% potassium ferricyanide (w v^−1^). The above mixture was incubated at 50 °C for 20 min. The solution was then cooled rapidly and 0.5 ml of 10% TCA (w v^−1^) was added. The mixture was then centrifuged at 3000 rpm for 5 min. One milliliter of the upper layer of the mixture was mixed with 1 ml of 0.1% FeCl_3_ (w v^−1^). The absorbance was noted after 10 min at 700 nm. Distilled water was used as blank for the experiment. Trolox was used as a standard for reducing power activity. A higher absorbance indicated higher reducing power. Trolox (0.5 mg ml^−1^) was used as the standard [31].

### DPPH free radical scavenging assay

The DPPH (2,2-diphenyl-1-picrylhydrazyl) assay is based on the scavenging of DPPH radicals leading to a decrease in the absorbance at 517 nm. The cell-free supernatant was 1:10 diluted with distilled water (D/W) before use. Around 40 μl of cell-free supernatant was mixed with 140 μl of methanol and 40 μl DPPH (0.15 mM). The absorbance was noted at 517 nm. Trolox (0.5 mg ml^−1^) was used as a standard in the assay [32].

The percentage inhibition was calculated by the following formula:

$$ \mathrm{DPPH}\ \mathrm{scavenging}\ \mathrm{activity}=\frac{\left(\mathrm{Absorbance}\right)t=0-\left(\mathrm{Absorbance}\right)\ t=15\ }{\left(\mathrm{Absorbance}\right)\ t=0}\times 100, $$

where *t*_0_ = absorbance at 0 min and *t*_15_ = absorbance after 15 min.

### ABTS free radical scavenging assay

The ABTS radical scavenging activity was measured 734 nm using Hitachi Spectrophotometer. The reagent was prepared by mixing 7 mM ABTS in water with 2.45 mM potassium persulfate to produce the radical. This mixture was allowed to stand for 16 h in the dark before use. The reagent was freshly prepared and used within 3 days. The ABTS stock solution was diluted with methanol and set to an absorbance of 0.7 ± 0.020 at 734 nm. The supernatants obtained after centrifugation were diluted (1:10) with methanol to obtain inhibition in the range of 20–95%. Trolox (0.5 mg ml^−1^) was used as standard for the assay. Forty microliters of test supernatant was mixed with 260 μl of ABTS solution and the absorbance was noted after 6 min at 734 nm. The lowest absorbance indicates the highest antioxidant activity. The percentage inhibition was calculated by the following formula:

$$ \mathrm{Percent}\ \mathrm{inhibition}=1-\frac{As}{Ac}\times 100; $$

where *A*_*c*_ = absorbance of control and *A*_*s*_ = absorbance of sample [33, 34].

### Statistical analysis

All the assays were performed in triplicate. The Statistical Package for Social Sciences (SPSS) software for Windows version (16.0) was used to compare the strains using analysis of variance (ANOVA). Post hoc tests like Duncan were approached to show the significance at *p* < 0.05.

## Results

### Qualitative detection of enzymes

All the enterococcal strains produced a zone of precipitation after adding iodine. The starch hydrolysis activity was displayed by all the enterococcal strains revealing amylase production (Table 1). Also, all the strains hydrolyzed Tween 80 by producing precipitation zones around the colonies indicating lipase activity. Gelatinase and cellulase activity was absent in the tested *Enterococcus* strains. Protease activity was displayed by all the strains by hydrolyzing casein. Strains *E. phoeniculicola* S20A, *E. dispar* S16B, *E. canintestini* AB1, and *E. dispar* S27A were bile-esculin positive. All the strains were hippurate non-hydrolyzing, urease and oxidase negative. The results can be related with *Enterococcus* spp. identification key [35].

**Table 1:** Qualitative study of hydrolysis of various substrates by *Enterococcus* strains

Strains	Starch	Tween 80	Cellulose	Gelatin	Casein	Esculin	Urea	Hippurate	Oxidase	β-galactosidase
AB2	+	+	–	–	+	–	–	–	–	–
SB2	+	+	–	–	+	–	–	–	–	–
S20A	+	+	–	–	+	+	–	–	–	–
S14B	+	+	–	–	+	–	–	–	–	–
AB1	+	+	–	–	+	+	–	–	–	–
S16B	+	+	–	–	+	+	–	–	–	–
S22C	+	+	–	–	+	–	–	–	–	–
S18A	+	+	–	–	+	–	–	–	–	–
SB3	+	+	–	–	+	–	–	–	–	–
S27A	+	+	–	–	+	+	–	–	–	–
S14B	+	+	–	–	+	–	–	–	–	–
S26B	+	+	–	–	+	–	–	–	–	–
S20B	+	+	–	–	+	–	–	–	–	–
LCS	+	+	–	–	+	–	–	–	–	–

+, hydrolysis; −, no hydrolysis

### Deconjugation of bile salts

The bile salt precipitation zone was measured and categorized accordingly. The precipitation zone is formed by the bile salt hydrolase (BSH) enzyme by breaking down the bile salt. All the strains displayed high BSH activity (Table 2).

**Table 2:** Bile salt hydrolase activity

Strains	Precipitation zone (mm)	BSH activity
SB3	16.7 ± 0.42	H
AB1	18.65 ± 0.49	H
SB2	18.3 ± 0.14	H
S18A	19.85 ± 0.21	H
S26A	15.2 ± 0.28	H
S20B	13.85 ± 0.21	H
S22C	19.3 ± 0.28	H
S14B	17.9 ± 0.24	H
S20A	17.2 ± 0.19	H
S14B	18.65 ± 0.36	H
S27A	17.8 ± 0.31	H
S16B	19.3 ± 0.26	H
SB2	19.4 ± 0.19	H
LCS	16.6 ± 0.37	H
*E.coli*	2.30 ± 0.21	L

Values reported are mean ± S.D. of three independent experimentsH; high (10–20 mm), M; medium (5–10 mm), L; very low/negligible activity (0–5 mm)

*H* high (10–20 mm), *M* medium (5–10 mm), *L* very low/negligible activity (0–5 mm)

### Production of biogenic amines

The strains were screened for the production of biogenic amines. All the enterococcal strains produced tyramine and agmatine after 3–4 days of incubation at 25 °C and 37 °C. Biogenic amine formation was not observed at 4, 12, and 20 °C (Table 3). Putrescine, tyramine, and cadaverine production was observed in the control strain *L. casei* Shirota. The results reveal that the amines formed only above 25 °C. Similar results were observed in the case to identify an enterococcal population in milk. The authors reported the presence of these genes *tyr* and *arg* in the enterococcal strains. They also found that these enzymes were specifically produced in protein-rich foods and also was particularly temperature dependent [6].

**Table 3:** Detection of biogenic amines

Strain	Amino acids					
	Lysine	Ornithine	Arginine	Histidine	Serine	Tyrosine
S20A	–^b^	–	+^a^	–	–	+
S27A	–	–	+	–	–	+
SB3	–	–	+	–	–	+
SB2	–	–	+	–	–	+
S20B	–	–	+	–	–	+
S26B	–	–	+	–	–	+
S22C	–	–	+	–	–	+
S16B	–	–	+	–	–	+
S18A	–	–	+	–	–	+
AB2	–	–	+	–	–	+
AB1	–	–	+	–	–	+
S14B	–	–	+	–	–	+
S26A	–	–	+	–	–	+
LCS	+	+	–	–	–	+

^a^Biogenic amine production detected above 25 °C after 4 days of incubation

^b^Not detected

### Protease, amylase, and lipase activity

**Fig. 1:**
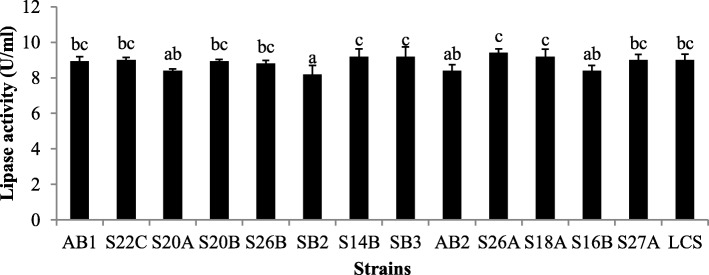
Lipase activity of the *Enterococcus* strains. The strains are AB1; *E. canintestini* AB1, S22C; *E. rivorum* S22C, S20A; *E. phoeniculicola* S20A, S20B; *E. dispar* S20B, S26B; *E. canintestini* S26B, SB2; *E. villorum* SB2, S14B; *E. rivorum* S14B, SB3; *E. canintestini* SB3, AB2; *E. canintestini* AB2, S26A; *E. dispar* S26A, S18A; *E. canintestini* S18A, S16B; *E. dispar* S16B, S27A; *E. dispar* S27A, LCS; and *L. casei* Shirota, respectively. Columns with different letters are significantly different *p* < 0.05

The proteolytic activity was displayed by all the strains in range of 192–264 mg ml^−1^. Strains *E. phoeniculicola* SB3 and *E. canintestini* S20A produced the highest amount of protease enzyme (264 mg ml^−1^) as compared to *L. casei* Shirota (200 mg ml^−1^). Amylase enzyme was also produced by all the strains (Table 4). The amylase enzyme was secreted by all the strains in the range of 0.5–0.7 mg ml^−1^. Strain *E. canintestini* AB1 exhibited the highest amylolytic activity (0.7 mg ml^− 1^) followed by *E. canintestini* AB2. The enterococcal strains were screened for their lipase activity by using tributyrin as lipase enzyme inducer. All the tested strains exhibited excellent lipase production in the range of 8–9 U ml^−1^. Strain *E. dispar* S26A showed the highest amount of enzyme activity of 9.4 U ml^−1^ greater than the *L. casei* Shirota strain. Figure 1 shows the lipase production of strains.

**Table 4:** Amylase and protease enzyme production by enterococcal strains

Strains	Amylase (mg ml^−1^)	Protease (mg ml^−1^)
S26A	0.6 ± 0.2^b^	212 ± 3.8^d^
S27A	0.54 ± 0.08^de^	200 ± 3.5^f^
SB2	0.56 ± 0.07^b^	208 ± 2.3^de^
AB2	0.63 ± 0.05^b^	240 ± 2.6^c^
SB3	0.6 ± 0.10^b^	264 ± 1.9^a^
S20A	0.56 ± 0.2^cd^	264 ± 2.5^a^
S26B	0.54 ± 0.09^de^	248 ± 1.9^b^
S22C	0.53 ± 0.06^e^	208 ± 2.4^e^
S16B	0.58 ± 0.12^bc^	240 ± 3.5^c^
S18A	0.58 ± 0.07^bc^	192 ± 3^g^
S20B	0.54 ± 0.2^de^	212 ± 3.4^d^
AB1	0.7 ± 0.08^a^	212 ± 2.5^d^
S14B	0.56 ± 0.06^cd^	204 ± 3.6^ef^
LCS	0.54 ± 0.15^de^	200 ± 2.8^f^

Values reported are mean ± SD of triplicate experiments. Values within each row and column with different letter assigned are significantly different *p* < 0.05

### β-galactosidase activity

The β-galactosidase activity was absent in the enterococci strains (Table 1). Not all enterococcal strains are capable of producing this important enzyme. The absence of these enzymes does not mean that they are not fit for probiotic use. However, the presence of enzymes like amylase, protease, and lipase may prove their usefulness in the GIT.

### Conjugated linoleic acid production

**Fig. 2:**
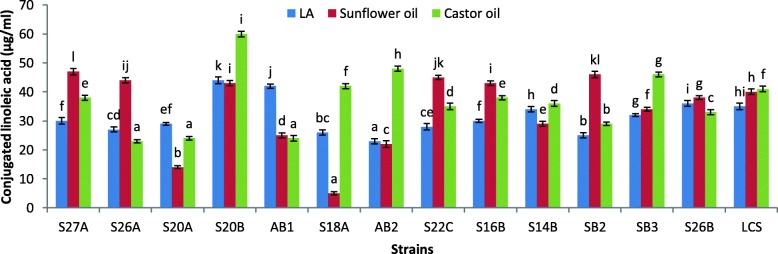
Production of conjugated linoleic acid from linoleic acid-rich substrates like sunflower oil and castor oil. Highest yield was seen in castor oil by strain S20B. Castor and sunflower oil both can be used as natural substrates for production of CLA

The production of conjugated linoleic acid from naturally rich substrates like castor oil and sunflower oil is graphically presented in Fig. 2. *E. dispar* S20B shows the highest conversion activity (60 μg ml^−1^) from castor oil. Strains *E. dispar* S20B, *E. rivorum* S22C, *E. dispar* S16B, *E. villorum* SB2, *E. canintestini* S26B, and standard *L. casei* Shirota produced ≥ 40 μg ml^−1^ from castor oil and sunflower oil. Kolaglu et al. reported about CLA production of about 33.5 ± 1.74 mg 100 g^−1^ by *L. lactis* subspp*. Lactis* and *L. reuteri.*

### Lactic acid production

**Fig. 3:**
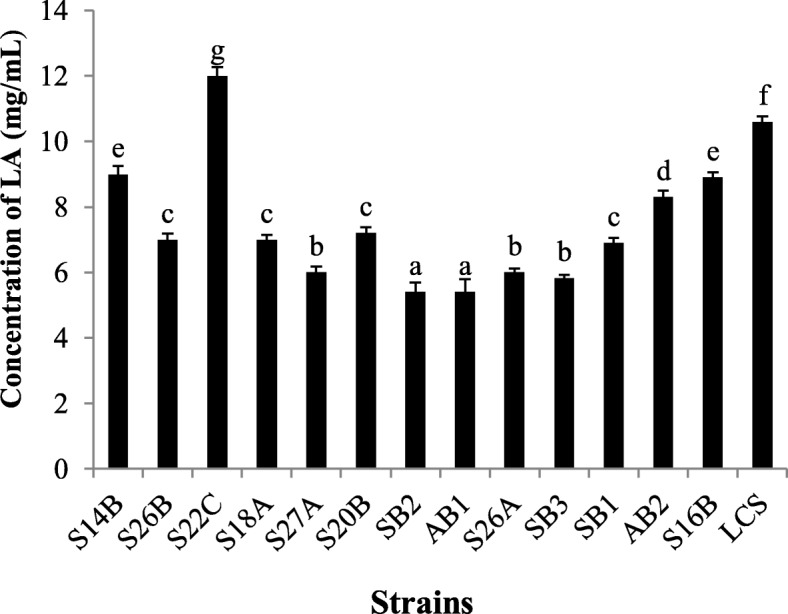
Quantification of lactic acid produced by *Enterococcus* strains by titrimetric method. Strain S22C revealed highest production (12 mg/ml) of lactic acid

The lactic acid production profile is elucidated graphically in Fig. 3. *E. rivorum* S22C shows the highest yield of lactic acid. The rest of the strains varied production values (7–10 mg ml^−1^).

### Antioxidant activity of *Enterococcus* strains

It is difficult to assess antioxidant activity by a single method owing to the irregularity of radical scavenging systems. Hence, it is suggested that one should determine the activity by using two or more methods. In this study, the radical scavenging property was assessed by DPPH, FRAP, and ABTS assays. Trolox, a known synthetic antioxidant, was used for comparing the activity of the strains in this study.

**Fig. 4:**
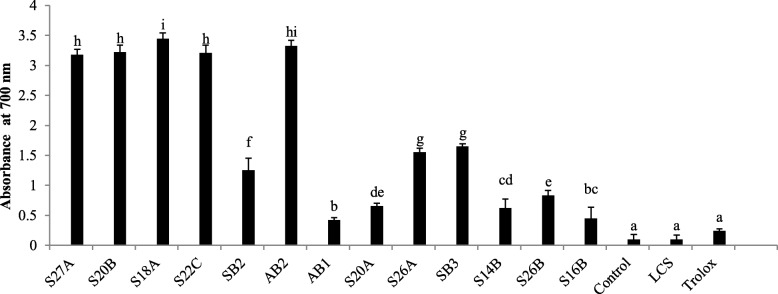
Total reducing power of *Enterococcus* strains cell-free supernatant by FRAP assay. The reduction activity of cell-free supernatants was measured by absorbance at 700 nm and compared with Trolox, a synthetic antioxidant. Columns with different letters are significantly different *p* < 0.05

The reducing power of extracellular extract was determined by using potassium ferricyanide. The reducers present in the test solution reduce the ferricyanide complex into the ferrous form. This reduction causes a change in color from yellow to blue or green. Figure 4 shows the reduction activity of the cell-free supernatant of strains. *E. canintestini* S18A showed the highest reducing power followed by *E. dispar* S27A, *E. dispar* S20B, *E. canintestini* SB3, and *E. dispar* S26A. Most of the strains showed desirable reducing properties with a significant difference (*p* < 0.05) as compared to the standard *L. casei* Shirota and Trolox.

**Fig. 5:**
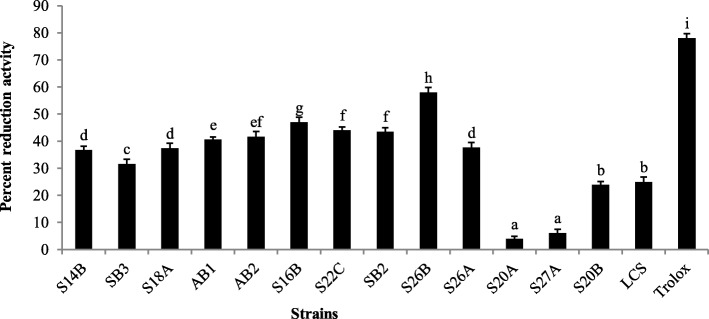
Antioxidant activity of *Enterococcus* cell-free supernatant by DPPH free radical scavenging assay. Columns with different letters are significantly different *p* < 0.05

The DPPH radical scavenging activity was explored for the cell-free supernatants. Figure 5 shows that strain *E. canintestini* S26B exhibited the highest scavenging activity of 57.98%; others (30–50%) while *E. dispar* S27A (6.146%) and *E. dispar* S27B (3.85%) exhibited the least activity. However, standard *L. casei* Shirota displayed 24.95% of radical scavenging property. The interaction of DPPH radicals with proton-donating antioxidants causes a decrease in the absorbance indicating scavenging property.

**Fig. 6:**
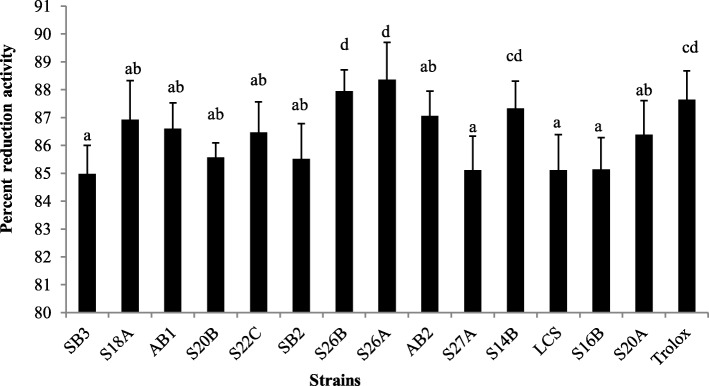
Antioxidant activity of *Enterococcus* cell-free supernatant by ABTS free radical scavenging assay. Columns with different letters are significantly different *p* < 0.05

The cell-free supernatants were screened for their ability to scavenge the ABTS radical. The strain *E. dispar* S26A showed the highest inhibitory activity (88.36%) against the radical (Fig. 6). All the tested strains and the standard strain displayed desirable inhibitory activity above 83%. Many strains exhibited higher activity with significant difference than *L. casei* Shirota (85.115%, *p* < 0.05) and were close to Trolox (87.64%) reduction activity*.* In this assay, ferryl myoglobin radicals are formed which lead to the oxidation of ABTS to ABTS^+^ radicals giving color. In the presence of an antioxidant, the reaction is suppressed to give a colorless solution [49].

## Discussion

The enzymatic activities of organisms play an important role in the food processing industry. The fermentation processes involve the secretion of various useful enzymes by the organisms. Hence, the screening of the bacteria for the enzyme production is necessary for food processing. Though the organisms produce useful enzymes, they also tend to produce harmful enzymes. Several lactic acid bacteria (LAB) used in the food industry are well-known for producing the desired enzymes. Hence, the qualitative analysis is of prime importance in sorting out the best organisms. The qualitative analysis sometimes gives false-positive results. Thus, biochemical assays serve in quantifying and detecting minute amounts of enzyme [36].

The bile hydrolysis is an important feature of the lactic acid bacteria. The cholesterol in the human body is stored in the form of conjugated bile salts and is excreted in the form of feces. Some fraction of these salts is not absorbed by the body and is released as free bile salts in the body. Thus, the deconjugation of bile salts can help in lowering the serum cholesterol levels in the lumen. The BSH enzyme produced by the bacteria helps maintain cholesterol levels in the body. Also, in order to survive the adverse conditions of the GIT, the bacteria produce bile salt hydrolase enzyme. These enzymes are produced as a defense mechanism by the bacteria against the bile salts [28]. Bile is secreted by the liver and attacks the bacteria by acting as detergents that dissolve the bacterial cell wall proteins. This is an intracellular enzyme and catalyzes bile salt hydrolysis. In a reported study, *E. faecium* EM 485 and EM 925 could hydrolyze bile salts in the medium [28]. Several lactic acid bacteria, as well as gut microbes, are reported to possess this enzyme activity [37]. The gut microbiota act on the intestinal bile by processes like deconjugation, dehydrogenation, deglucoronidation. Thus, the bile tolerance of the probiotic bacteria can be elucidated by the presence of this enzyme and it becomes a necessity factor of probiotic selection procedure [38].

Biogenic amines are generally found in poultry products, fish, meat, eggs, and fermented products like cheese and pickles. They are nitrogenous compounds produced by the body to confer physiological function of the immune response, brain, cell division, etc. But when these compounds when consumed orally, they prove to be toxic when taken in large quantities. Several researchers have reported the role of lactic acid bacteria in the production of biogenic amines. Thus, there is a possibility of production of these toxins during food processing. But, the production of these toxic amines takes place only after the bacteria has sought favorable conditions during processing and storage. The bacteria produce the decarboxylases only when free amino acids or protein-rich foodstuff are available. Thus, the development and modification of these parameters and vigilant screening of good bacterial strains might help in eradicating the involvement of such toxic metabolites [39, 40].

Amylase enzyme is necessary for the degrading of starch molecules, thus helping in smooth digestion. *Lactobacillus* cultures were reported to produce this enzyme in large quantities in the gastrointestinal transit in chickens. This enzyme is produced both intracellularly as well as extracellularly [42]. The proteolytic activity of *Enterococcus* is present in the extracellular component of the bacteria. This activity is attributed to the metalloproteinase activity that hydrolyzes casein in the milk. Several studies have reported the presence of this enzyme in large quantities during ripening of cheese. The inoculum dose of the bacteria or the enzyme must be controlled in the process of dairy products as greater amounts of enzyme lead to flavor and aroma defects in the products [36]. Proteases and lipases help in biofilm formation of the probiotic organism, thus inhibiting the pathogens from the GIT [41]. In a recent study presenting synergistic effects of lipase and enterocin production, EF-5653 showed maximum activity of 6 U ml^−1^ and EF-64 with 4 U ml^−1^ whereas standard *E. faecium* M5153 had no lipase activity [43]. All the strains reported in the present study showed excellent lipase activity when compared in the literature. This study reported the highest amounts of lipase activity by *Enterococcus* strains. The food industry involves the usage of lipases in the processing of cheese, dairy products, etc. The lipases secreted by these organisms also impart flavor and aroma to the product [42]. Certain studies have revealed the lethal effect of lipases on pathogens. The lipases acting synergistically with bacteriocins attack the lipid membrane of the bacterial cell, thus dissolving and helping in cell death [43].

Safflower oil, rice bran oil, palm oil, castor oil, sunflower oil, and soybean oil containing linoleic acid are natural sources of linoleic acid. Castor oil (90% ricolenic acid) and sunflower oil (at least 69%) are known for their rich linoleic acid content [44, 45]. Hence, these oils were selected and examined for their biochemical conversion of linoleic acid by enterococci. This is the first study to report excellent CLA production by enterococci using natural substrates. Hosseini et al. have reported the use of these substrates for CLA conversion using *L. plantarum* [46]. The addition of Tween 80 to the substrate may aid in lipase production which in turn frees linoleic acid from the oils, thus hastening the process of biochemical conversion of CLA. Several studies using *Lactobacillus*, *Bifidobacterium*, and *Propionibacterium* reveal that these bacteria are known to possess linoleic acid isomerase which converts ricolenic and linolenic acid to CLA isomers [12].

Lactic acid is a frequently used chemical in the food industry, a flavoring agent, preservative, and acidulant. It also has application in the textile and pharmaceutical industry [47]. Lactic acid production by microorganisms is a mostly favored process than the chemical one as it prevents environmental pollution [48]. Fowoyo and Ogunbanwo carried out a similar study involving five *Lactobacillus* strains of which the highest quantity of lactic acid (8 g l^−1^) was produced by *L. lactis* spp. [15].

All the strains showed higher reduction activity both by DPPH and ABTS assay when compared with the *L. casei* Shirota strain. In a similar study, *E. durans* LAB 18s strain exhibited high ABTS (9.4%) and DPPH activity EC_50_ = 3.6, respectively [50]. Pieniz et al. also reported the antioxidant activity of *E. faecium* strains (ABTS = 59–92.5%; EC_50_ = 2.41–5.02 μg/ml) isolated from meat and dairy products [51]. The consumption of radical scavenging probiotics has proven to be helpful in boosting the immunity of the host. The oxidative stress is caused by infectious agents and disorders which harm and deteriorate the cells. The probiotic bacteria are known to possess this activity. This attribute is contributed by the secondary antimicrobial compounds produced by the probiotic strain. The antioxidant activity of the probiotic bacteria helps in reducing the aging process, ulcers, cardiovascular disease, diabetes, and urinary tract infections. Recently, markets are flooded with synthetic carcinogenic antioxidants. Though these antioxidants help in slowing down the oxidation process, they may pose as a threat to the host. Consumption of bio-therapeutic microorganisms can scavenge the ROS radicals and save the body from its ill-effects. The cell-free supernatants of lactic acid bacteria have already proved their scavenging properties and thus have reduced the risk of chronic diseases [52]. The scavenging property of probiotics can be attributed to metal chelation, ROS radical scavenging, or ascorbate auto-oxidation activity [53]. The ROS theory proposes the development of antioxidative agents that would help in curbing the progression of free radical-related disorders.

## Conclusion

The present investigation revealed enzymes and secondary metabolites of therapeutic as well as industrial value. The screening of metabolites produced by indigenous flora also helps in studying the intestinal metabolism and interaction of the microbiota. Excellent antioxidant activities of *E. canintestini* S26B and *E. dispar* S26A proved the scavenging potential of these strains. They can be used along with other well-known probiotic strains after clinical trials. The production various enzymes like protease and lipase promise value of these strains in dairy and food-beverage fermentation industries. Their property to produce conjugated linoleic acid using castor oil shows its future potential in production and use of these metabolites in nutrition studies. Further studies will involve optimization and production of important industrial enzymes and specific type of CLA produced by enterococci strains along with in vivo studies to confirm their probiotic potential.

## Data Availability

Not applicable.

## Abbreviations

ABTS: 2,2-Azino-bis (3-ethylbenzothiazoline-6-sulphonic acid) diammonium salt
AOAC: Association of Analytical Communities
BA: Biogenic amines
BHI: Brain Heart Infusion broth
BSH: Bile salt hydrolase
CLA: Conjugated linoleic acid
DPPH: 1,1-Diphenyl-2-picrylhydrazyl
FAO: Food and Agriculture Organization
FeCl_3_: Ferric chloride
FRAP: Ferric reducing antioxidant power
*L. casei* YIT 9029: *Lactobacillus casei* Shirota
LAB: Lactic acid bacteria
MRS: Mann-Rogosa-Sharpe
NaOH: Sodium hydroxide
ONPG: O-nitrophenol galactosidase
PBS: Phosphate-buffered saline
ROS: Reactive oxygen species
TCA: Trichloroacetic acid
